# Hepatitis C Virus 5′UTR Sequences That Bind eIF3 and Ribosomal 40S Subunits Confer Stimulation of Minus-Strand RNA Synthesis

**DOI:** 10.3390/ijms27073234

**Published:** 2026-04-02

**Authors:** Attiya Qadoos Malik, Lyudmila Shalamova, Mozhdeh Khajouei, Jonas Budnik, Anna-Lena Hell, Elena Jost, Gesche K. Gerresheim, Oliver Rossbach, Michael Niepmann

**Affiliations:** 1 Institute of Biochemistry, Faculty of Medicine, Justus-Liebig-University, 35392 Giessen, Germany; qadoos@gmx.de (A.Q.M.); lyudmila.shalamova@vetmed.uni-giessen.de (L.S.); khajoueimozhdeh@gmail.com (M.K.); jonas.budnik@gmail.com (J.B.); anna-lenahell@gmx.de (A.-L.H.); jost.elena@web.de (E.J.); gesche.gerresheim@gmx.de (G.K.G.); 2 Institute for Virology, Faculty of Veterinary Medicine, Justus-Liebig-University, 35392 Giessen, Germany; 3 Department of Anesthesiology, Intensive Care Medicine and Pain Therapy, University Hospital Giessen, Justus-Liebig-University, 35392 Giessen, Germany; 4 Medical Faculty, RWTH Aachen University, 52074 Aachen, Germany; 5 University Children’s Hospital, University Medical Center Hamburg-Eppendorf, 20246 Hamburg, Germany; 6 Institute for Virology, Philipps-University Marburg, 35043 Marburg, Germany; 7 Institute of Biochemistry, Faculty of Biology and Chemistry, Justus-Liebig-University, Heinrich-Buff-Ring 17, 35392 Giessen, Germany; oliver.rossbach@chemie.bio.uni-giessen.de

**Keywords:** plus-strand, positive-strand, negative-strand, replication, encapsidation, packaging

## Abstract

Hepatitis C Virus (HCV) is a plus-strand RNA virus that replicates its genome via a minus-strand intermediate, which in turn is the template for the synthesis of progeny plus-strand genomes. In order to characterize sequence elements in the HCV 5′-untranslated region (5′UTR) that are possibly involved in the regulation of minus-strand RNA synthesis starting at the genome’s 3′end, we used a replicon system in which a possible function of these sequences is uncoupled from other functions like translation regulation. For the specific detection by RT-qPCR of minus strands newly synthesized in the cells from the transfected replicon RNAs, we carefully eliminated the contaminating DNA and transfected RNA and avoided self-priming caused by hairpin formation. We found that the absence of any HCV sequences at the 5′end does not allow genome replication. Stem-loop I-II sequences only allow extremely low-level replication, whereas the presence of stem-loops I-III or the complete 5′UTR allows efficient replication. The mutation of sequences required for the binding of translation initiation factor 3 (eIF3) and the ribosomal 40S subunit in the 5′UTR of the plus strand severely impairs minus-strand synthesis. This suggests that eIF3 and the 40S subunit are involved in plus-strand 5′-3′-end communication and the regulation of minus-strand synthesis.

## 1. Introduction

HCV mainly infects liver cells and can cause liver cirrhosis and liver cancer. The genome of HCV is a plus-strand RNA of about 9600 bases [1,2] that replicates via a minus-strand antigenome [3,4,5,6,7]. The 5′end of the plus strand has no standard m^7^G Cap but often contains an FAD Cap [8] as protection against exonucleases, and translation of the viral polyprotein is started by an Internal Ribosome Entry Site (IRES) element [9,10]. This arrangement has the advantage that both extreme ends of the RNA genome (on the plus and on the minus strand) are available for replication-active RNA *cis*-signals. Several RNA secondary structures in the plus strand are involved in the regulation of viral RNA replication; these structures are located largely at the ends but also in the internal regions of the RNA genome [2,5,7,11,12,13,14,15,16,17,18]. To a minor extent, functional RNA secondary structures are also present at the end of the minus strand [2,16,19,20,21,22,23,24].

**Figure 1:**
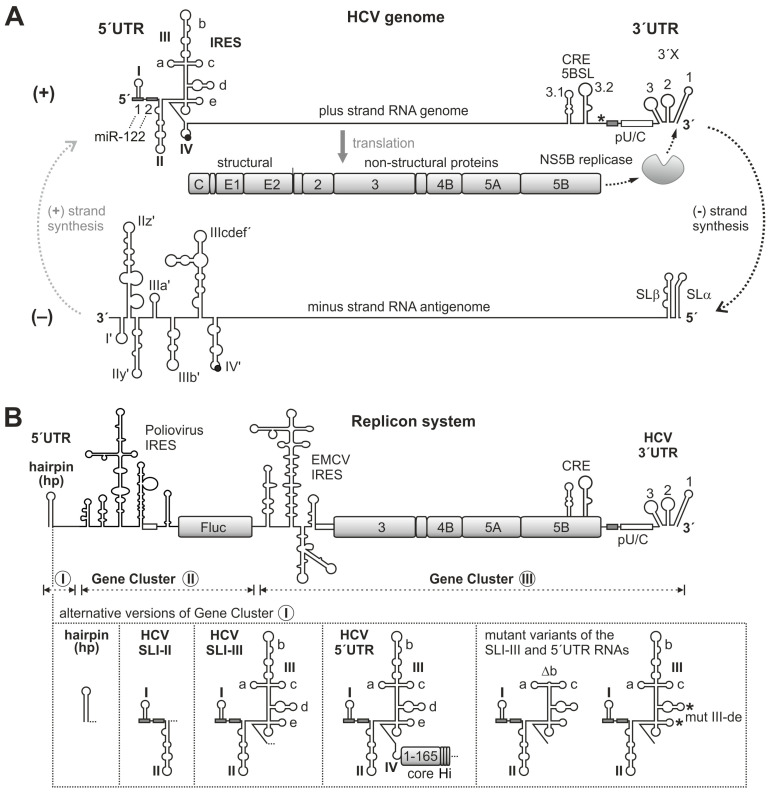
HCV genome replication and the replicon system. (**A**) The HCV plus (or “positive”)-strand RNA genome with the 5′ untranslated region (5′UTR), the polyprotein coding sequence (CDS) and the 3′UTR. In the 5′UTR, the RNA secondary structure stem-loops (SLs) II–IV constitute the internal ribosome entry site (IRES) element that starts the translation of the polyprotein at the AUG (black dot) in SLIV. The 3′UTR downstream of the CDS stop codon (*) contains a polyU/C-tract (pU/C) and conserved stem-loops at the end. Binding sites for microRNA-122 (miR-122) are indicated by gray boxes. The non-structural (NS) protein NS5B is the RNA-dependent RNA polymerase (RdRP or “replicase”) that starts the synthesis of the minus (or “negative”)-strand RNA at the 3′end of the plus strand. In the NS5B coding sequence, a *cis*-replication element (CRE) is required for replication. The minus strand has RNA secondary structures in its 5′- and 3′-regions that do not exactly mirror those in the plus strand due to different G-U base pairs. Progeny plus-strand RNA genomes are then replicated from the minus strand starting at its 3′end. (**B**) The HCV RNA replicon system contains three independent gene clusters (I, II and III). In Gene Cluster III, an Encephalomyocarditis Virus (EMCV) IRES drives expression of the NS3–NS5B proteins that are required for RNA genome replication, and the CRE and the HCV 3′UTR provide *cis* signals involved in the start of minus-strand RNA synthesis. In Gene Cluster II, the Poliovirus IRES drives the translation of an independent reporter, firefly luciferase (Fluc). At the 5′end of the replicon RNA, Gene Cluster I contains either a strong stem-loop (“hairpin”) that protects the RNA against degradation, or various lengths of the HCV 5′UTR. In the complete 5′UTR version, the HCV IRES initiates the translation of the C-terminally truncated HCV Core protein with a triple HiBiT tag (Hi). In the SLI-III or complete 5′UTR versions of this Gene Cluster I, a deletion of SLIIIb (ΔIIIb) impairs the binding of eIF3, or mutation of SLs IIId and IIIe (*, mutIIId/e) impair the binding of the small ribosomal 40S subunit.

Replication of the HCV plus-strand RNA starts at the 3′end of the 3′UTR (Figure 1A), which has a very characteristic organization. The polyprotein stop codon is followed by a binding site for the liver-specific microRNA-122 (miR-122), a polyU/C tract and the highly conserved so-called 3′X region [25,26]. The 3′X region comes in two possible conformations, one containing three stem-loops (counted from the 3′end SL1, SL2 and SL3), or SL2 and SL3 can refold to form one longer stem-loop that is suspected to act as a dimer linkage sequence (DLS) for genome dimerization [5,27].

In addition to *cis*-elements in the 3′UTR, two RNA secondary structures upstream of the NS5B stop codon [2,7] are also required for replication. These comprise the so-called *cis*-replication element (CRE) with the 5BSL3.2 (also called SLV) and the upstream 5BSL3.1 (SLVI) [28,29]. The CRE is also involved in long-range RNA-RNA interactions (LRIs) with the 3′UTR and the 5′UTR [2,7,30,31]. The most prominent of these LRIs is the interaction of the apical loop of the CRE/5BSL3.2 with the SL2 of the 3′UTR, also called a “kissing-loop” interaction [32,33].

To start minus-strand synthesis, the NS5B replicase needs to bind to the HCV 3′UTR and start the synthesis of the minus strand [34]. In biochemical activity assays that come in a setting with reduced complexity, the start of RNA synthesis by NS5B can occur on the 3′end of a single-stranded RNA template. In this context, NS5B has a preference for GTP as a starter nucleotide [35,36]. After a slow initiation process with the incorporation of the first two or three nucleotides, beginning with the next nucleotide, the K_M_ for nucleotide incorporation drops sharply, indicating a transition from an inefficient initiation mode to an efficient elongation mode [35,37,38]. Thereby, the polymerase can also use the 3′-terminal U of the template to incorporate FAD as a starter nucleotide [8,39].

However, the interaction of the NS5B replicase with its genuine RNA template comes with several different aspects and appears rather complicated. The formation of the strong stem of SL1 at the very 3′end of the RNA genome (see Figure 1A) is highly conserved [2] and required for efficient replication. Mutations affecting this stem, including the mutation of the terminal U residue that can be involved in a G-U base pair, impair replication [13,40]. In in vitro assays in the absence other HCV replication protein functions, the 3′end of the plus strand is a much worse template for the NS5B polymerase than the 3′end of the minus strand [41]. Nevertheless, the replicase obviously needs to bind to the 3′-terminal SL1, and this stem must be unwound to allow NS5B to access the 3′end of the plus strand. In HCV replication complexes, the unwinding of SL1 likely takes place due to the helicase activity of NS3 [42]. Likely compensating for this inefficient template situation, the NS5B protein also binds to sequences in the upstream NS5B coding region. There, the CRE 5BSL3.2 and the 5BSL3.1 both bind NS5B [29,43], with a stronger contribution of 5BSL3.2 [29]. Also downstream of the stop codon, NS5B can bind to polyU and to the SL2 of the 3′X region [29,34,43,44]. Thereby, the affinity of the replicase to the RNA regions like the 5BSL3.2 or the polyU is higher than its affinity to the 3′X region [44,45], although the latter contains its genuine RNA synthesis start position.

Thus, the 3′end of the HCV plus-strand RNA genome is—on first glance—evolved to be a “difficult” template for its polymerase, since obviously melting the strong stem of SL1 is required in order to bind the very 3′terminal nucleotide of the template strand. Therefore, besides the contribution of the NS3 helicase function in melting the SL1 [42], the formation of dimers or oligomers by the NS5B protein [46,47,48,49,50,51,52] might support a multi-point interaction of NS5B with upstream RNA elements that stabilize NS5B binding to its genuine template. In this context, the pausing of two ribosomes with nearly completely folded NS5B proteins at the NS5B stop codon and on conserved slow codons directly upstream may support both NS5B dimerization and its search for the multiple binding sites on its own template RNA in *cis* [52,53]. This multi-point tethering of some NS5B molecules in the oligomer to higher-affinity binding sites may then allow another monomer of the NS5B oligomer to interact more transiently with the actual start site in *cis*. These more transient interactions with the actual start site may be required for an escape of the polymerase from the transcription start site and the transition from initiation to elongation (“promoter escape”). Finally, this whole complicated setting may serve to make the HCV 3′end available as a substrate only to the HCV replication machinery in *cis* and not to other (cellular) polymerases. In turn, this may ensure the specificity of the HCV replication machinery only for its genuine viral template.

Considering the polymerase starting RNA synthesis at the RNA genome’s 3′end, two major aspects appear evident. On the one hand, a collision between ribosomes translating the plus-strand RNA in the 5′- to 3′-direction with the polymerase synthesizing the minus strand in the opposite direction should be avoided. This scenario would require a mutual negative regulation of translation versus replication. On the other hand, in analogy to the Cap-polyA interaction in cellular mRNAs, a 5′-3′-interaction in viral genomes should ensure that only intact, undegraded viral RNA genomes are both efficiently translated and efficiently replicated. Therefore, it makes sense that the HCV 3′UTR stimulates translation directed by the 5′UTR IRES (ref. [54] and references therein). Vice versa, it also makes sense that only intact, undegraded viral RNA genomes are worth being replicated efficiently. Thus, it can be suspected that in this scenario, sequences at the 5′end might stimulate the initiation of minus-strand RNA synthesis at the genome’s 3′end in *cis*.

In this study, we therefore aimed to identify sequences in the 5′UTR of the HCV plus strand that regulate minus-strand synthesis via communication between the 5′- and 3′ends of the plus strand. In principle, in experiments addressing such a question, the molecular process under investigation (here: minus-strand RNA synthesis) needs to be uncoupled from all other processes like translation, plus-strand synthesis, genome packaging, virion assembly and so on. In comparison, the few studies that fulfill the above uncoupling requirements for the investigation of the start of plus-strand RNA synthesis at the 3′end of the minus strand have used a two-component in vitro system with (1) a purified RNA template representing only the 3′end of the minus strand and (2) a recombinantly expressed and purified NS5B polymerase, with the readout being newly synthesized short pieces of plus-strand RNA [21,23,24]. These studies showed that certain sequences and RNA secondary structures at the 3′end of the minus strand facilitate the plus-strand RNA synthesis initiation by NS5B. In contrast, other studies that investigated determinants at the 5′end of the annotated genome that are involved in the regulation of RNA synthesis [12,14,15,16] used subgenomic HCV replicon systems that contain both genome ends, usually measuring plus-strand genome abundance as a readout for replication capability. However, this readout is generated by the intracellular replication of the replicon RNA, which involves repeated rounds of minus- and plus-strand synthesis (referred to as “roundabout” amplification of replicon RNA).

However, such replicon systems capable of “roundabout” amplification come with two intrinsic properties that—strictly speaking—preclude an easy and unambiguous assignment of cause–effect relationships. These two challenges may sound similar on first glance but they are different: (1) these replicon systems contain *cis* elements at both genome ends, and it appears difficult to clarify if a sequence under investigation acts on the genome’s 5′end function, or if it acts (possibly by long-range interactions) on the functionality of the genome’s 3′end in replication. (2) It is even more difficult to find out by genetic experiments if a *cis*-element under investigation actually exerts its function on the physical level of the plus strand or on the physical level of the minus strand. For example, mutations that are inserted in a double-stranded DNA plasmid that serves as a transcription template for a plus-strand replicon RNA may have been planned to affect the function of the stem-loop I close to the 5′end of the plus strand (Figure 1A). However, we must ask the question of whether the mutation actually affects the function of the SLI in the plus strand, or if the mutation actually affects the function of the reverse complement SLI’ element at the 3′end of the minus strand, or both. This intrinsic logical dilemma with overlapping *cis* elements on both opposite strands is the reason why some studies investigated the function of RNA secondary structure elements at the 3′end of the minus strand in in vitro systems of reduced complexity [21,23,24].

In the absence of a fully functional in vitro system for the investigation of minus-strand synthesis initiation at the 3′end of the plus strand, the above problems regarding the unambiguous assignment of cause–effect relationships can be avoided at least partially by (1) using a replicon system in which the possible function of 5′UTR sequences in regulating the efficiency of minus-strand RNA synthesis at the genome’s 3′end is uncoupled from other functions, and by (2) measuring the abundance of the direct product of the function under investigation, i.e., minus-strand abundance.

Therefore, regarding point (1), we developed a replicon system that comes with the HCV 5′UTR sequences completely uncoupled from all other functions, and in its basic form it actually has no HCV 5′UTR sequences (Figure 1B). Regarding point (2), we used RNA purification and RT-qPCR for the specific detection of only HCV minus-strand RNAs. In fact, the latter point proved by far to be more difficult than expected; in particular, the removal of contaminating DNA and unwanted RNA degradation products required a largely improved protocol for RNA purification.

Our results show that (1) replicons lacking any HCV sequences at the 5′end are incapable of replication, (2) in contrast to previous reports [12,14,16], replicons only containing the HCV 5′UTR stem-loops I and II replicate only on an extremely low level, (3) the presence of 5′UTR sequences including SLs I-III or the complete 5′UTR allows efficient minus-strand synthesis, and (4) sequences in the 5′UTR that are required for the binding of the translation initiation factor eIF3 and the ribosomal 40S subunit stimulate RNA synthesis. These results indicate that eIF3 and the 40S ribosome play an important role not only in translation directed by the HCV IRES, but also in 5′-3′end communication and the regulation of minus-strand synthesis.

## 2. Results

### 2.1. A Replicon System for the Detection of Uncoupled HCV Minus-Strand Synthesis

In order to investigate a possible contribution of HCV 5′UTR sequences to the regulation of minus-strand synthesis, we developed a replicon system that contains three Gene Clusters (Figure 1B, for details please see Section 4). Gene Cluster I contains the HCV 5′UTR sequences under investigation or only a hairpin (hp) as a negative control. Gene Cluster II provides an independent expression cassette for the indirect quantification of replicon RNA abundance by virtue of *Firefly* luciferase (Fluc) expression under Poliovirus IRES control. Gene Cluster III contains the EMCV IRES driving expression of the HCV NS3 to NS5B replication proteins, followed by the HCV 3′UTR as the platform for launching minus-strand RNA synthesis. By this arrangement, a potential role of HCV sequences at the genome’s 5′end in regulating RNA synthesis at the genome’s 3′end is completely uncoupled from the expression of the HCV replication proteins and other functions.

### 2.2. Improvement of Specific Detection of HCV Minus Strands

The reliable quantification of minus strands as the direct product of the function under investigation required a considerable improvement of RNA purification and detection methods. Starting from the in vitro synthesis of the plus-strand replicon RNA to be transfected through to the final RT and qPCR reactions for detection of newly synthesized minus strands, the entire procedure comprises a series of optimized methods that were carefully adapted to ensure highly accurate measurement of minus-strand abundance.

**Figure 2:**
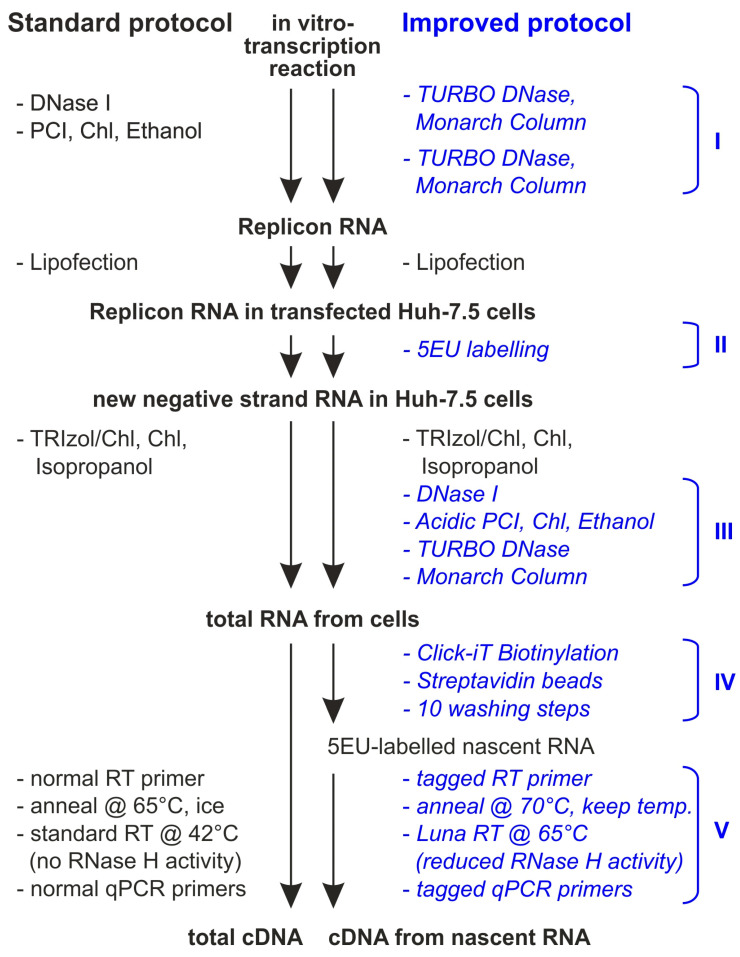
Improved RNA purification before and after transfection of HCV replicon RNA. On the left, standard procedures (black) are shown for RNA purification after in vitro transcription and during isolation of RNA from transfected cells. On the right, five phases of improved procedures (blue) are shown. In general, after RNA isolation from cells using standard TRIzol purification, five additional purification steps with four different physico-chemical mechanisms of separation were applied, namely enzymatic degradation (DNase and TURBO DNase), aqueous/organic partitioning (with acidic phenol), salt-mediated polar interactions (with Monarch silica matrix columns) and affinity purification of the biotinylated RNA. In particular, labeling of RNA newly synthesized in the cells with 5-Ethynyl-Uridine (5EU) after transfection of the replicon RNA (phase II) allows biotinylation of that RNA, followed by purification on streptavidin beads (phase IV). Replicon RNAs isolated in this way were used for detection on minus strands, whereas plus strands were detected after improved RNA isolation but without purification using streptavidin beads. During annealing of a tagged primer for reverse transcriptase (RT) and qPCR reactions, high temperature (70 °C/65 °C) was applied without interruption. This helps to reduce self-priming of the hairpin at the HCV 3′UTR 3′end, as well as unwanted priming by copurified residual RNA and DNA oligonucleotides that were generated by degradation. PCl: phenol/chloroform/isoamylalcohol (125:25:1) treatment; Chl: chloroform treatment; Ethanol: ethanol precipitation and washing.

In comparison to standard procedures, five parts of the procedure were largely improved (Figure 2): (I) the refined removal of residual plasmid template DNA after in vitro transcription, (II) the addition of 5-Ethynyl-Uridine (5EU) to the cells to only label RNA newly synthesized after replicon transfection, (III) the extremely careful DNA removal after RNA isolation from cells, (IV) the specific enrichment of only 5EU-labeled RNA that was newly synthesized after transfection, and (V) primer annealing RT and qPCR reactions were performed under conditions of uninterrupted high temperature, as well as the use of tagged primers to improve the specificity of target detection [55].

Before we applied these improvements, the background of the RT-qPCR reactions with replication-defective constructs (Pol −) was up to 25% of the 5′UTR WT construct. This was likely due to a variety of problems that generate false–positive signals with any of the replicon RNA constructs, including the polymerase-deficient negative controls. These problems are: (1) partially degraded residual template plasmid DNA oligonucleotides could hybridize to the in vitro transcribed RNA and later act as unwanted RT primers. (2) Minute amounts of longer plasmid DNA pieces could even serve as qPCR templates on their own. (3) The SL1 hairpin of the HCV 3′UTR can give rise to self-primed copy-back synthesis of minus-strand RNAs by the T7 RNA polymerase, already using the freshly made RNA product as a template during the in vitro transcription (ref. [56] and references therein). (4) In the transfected cells, partially degraded RNA oligonucleotides from regions of RNA secondary structures, derived from the large amounts of transfected replicon plus-strand RNAs, can hybridize to residual plus-strand pieces and act as unwanted primer/template combinations in the RT reaction. (5) In the RT reaction, the SL1 hairpin at the HCV 3′UTR 3′end can give rise to reverse transcription synthesis of minus-strand sequences in any replicon construct.

After applying the above improvements, the background in the negative controls dropped to about 2.5% at early time points (4 and 24 hpt, hours post-transfection) and to about 0.025–0.03% at later time points (72 hpt, also see Figures below). The higher background signals at early times after transfection are likely due to residual partially degraded RNA primer/template combinations generated from the transfected plus strands (the above point 4). These RNAs are subject to ongoing degradation in the cells after transfection, and therefore the background drops with increasing time after transfection. Perhaps such background could be further reduced by washes of the biotinylated minus-strand RNAs on the streptavidin beads with wash buffers that contain reagents stripping off RNA from RNA, like urea or guanidinium, plus EDTA.

### 2.3. Contribution of HCV 5′UTR Sequences to HCV RNA Genome Replication

**Figure 3:**
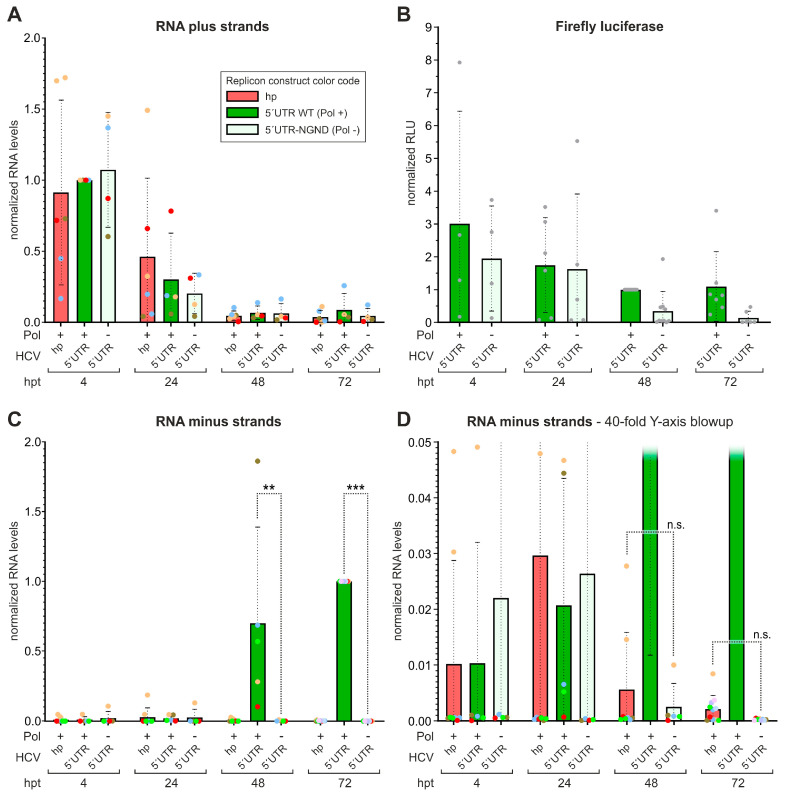
Detection of HCV replicon minus-strand RNA synthesis. In vitro transcribed RNAs of three replicon constructs were transfected into Huh-7.5 cells. The “hp” construct has only a protective stem-loop at its 5′end (“Gene Cluster I”, see Figure 1B), while the “5′UTR” constructs have the complete HCV 5′UTR. The latter come with either wild-type NS5B polymerase activity (WT; Pol +), or with the NGND mutation in the NS5B active center (Pol −). At 4, 24, 48 and 72 h after transfection (hpt), cells were either lysed and the cell extracts were used for RNA isolation and detection (**A**,**C**,**D**), or separate cell lysates were prepared for firefly luciferase (Fluc) measurement (**B**). The same construct color code is used through all panels. (**A**) Detection of HCV replicon RNA plus strands by specific strand-specific RT-qPCR. Values show means and standard deviations (SDs), dots show values of individual experiments. (**B**) Firefly luciferase (Fluc) activity expressed from Gene Cluster II as an indirect measure for replicon RNA plus-strand abundance. (**C**) Detection of HCV replicon minus strands by strand-specific RT-qPCR after 5EU labeling, biotin linkage and streptavidin bead purification of RNA newly synthesized in the Huh-7.5 cells after replicon RNA transfection (see improved protocol in Figure 2). (**D**) 40-fold magnification (“blowup”) of Y-axis from (**C**) to show low-value details. At 72 hpt, the 5′UTR-NGND (Pol-) replicon showed 0.025% of the 5′UTR WT (Pol +), with an SD of 0.014%. Statistical significance for pair-wise comparisons is shown with ** *p* < 0.01; *** *p* < 0.001; and *p* > 0.05 not significant (n.s.).

First, the replicon with the complete HCV 5′UTR in Gene Cluster I was used, in the form with replication-competent NS5B polymerase (Pol +) or with the NGND mutationdisabling polymerase activity (Pol −). For comparison, the basic “hp” construct was used in which the 5′-terminal HCV sequences were replaced by a protective strong stem-loop (see Figure 1B). The transfected plus strands of different constructs were degraded over time equally well (Figure 3A). Thus, differential degradation of the transfected plus-strand RNAs is not supposed to give rise to differences in minus-strand synthesis between constructs. Moreover, this also indicates that the hairpin at the 5′end of the “hp” replicon protects that RNA as well as the others are protected by the genuine HCV 5′UTR RNA secondary structures. *Firefly* luciferase activity was used as an indirect measure for plus-strand abundance of the 5′UTR replicons (Figure 3B). Also here, at earlier time points (4 and 24 hpt), both NS5B polymerase-competent and -defective constructs expressed Fluc equally well, consistent with the presence and translation of the transfected plus-strand RNA detected in Figure 3A. While Fluc activity is fully maintained in polymerase WT constructs at later time points, it declines in Pol-deficient constructs. This suggests that the transfected RNA is further degraded, and Fluc is expressed further only from those constructs with an active NS5B polymerase that replicate and produce progeny plus strands.

Minus-strand-specific RT-qPCR (Figure 3C) shows the detectable production of minus strands after 48 hpt and later. In the magnification in Figure 3D, we see that the background caused by primer/template combinations derived from degraded plus strands declines with time, reaching about 0.025% at 72 hpt (see technical discussion in Section 4). Importantly, the hp construct is negative in this minus-strand assay. This means that HCV RNA replication is not possible without a genuine HCV sequence at the 5′end of the annotated genome. However, at these late time points, it is not possible to decide at which stage of RNA replication the missing sequences would be required, and it is likely that they can also act at the 3′end of the minus strand [21,23,24].

**Figure 4:**
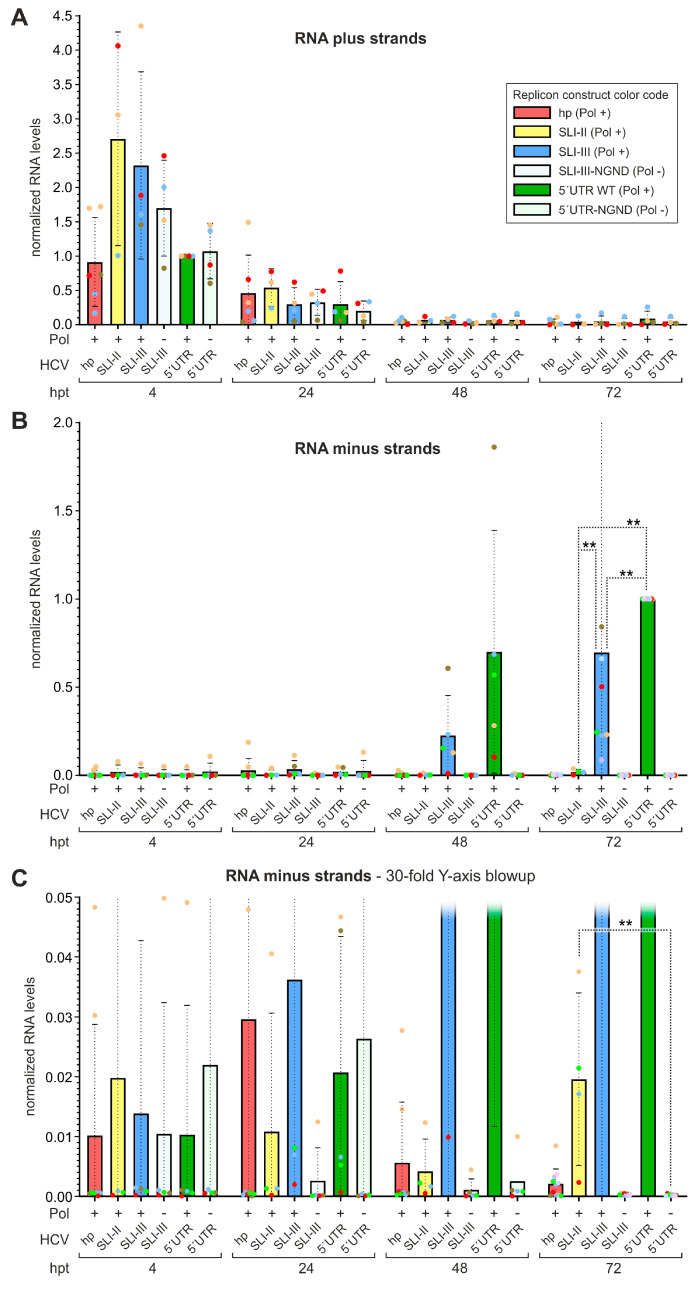
Minus-strand synthesis proficiency of HCV replicon RNAs with different lengths of 5′UTR sequences. Replicon constructs contain either the hairpin (hp) or HCV 5′UTR sequences as shown in Figure 1B, lower panel, with the NS5B replicase active (Pol +) or inactive (Pol −). The experiment was performed as shown in Figure 3, and the same construct color code is used through all panels. (**A**) Strand-specific RT-qPCR detection of plus strands. (**B**) Strand-specific RT-qPCR detection of 5EU labeled minus strands. (**C**) 30-fold magnification of Y-axis from (**B**) to show low-value details. At 72 hpt, the SLI-III-NGND (Pol −) replicon showed 0.032% of the SLI-III WT (Pol +), with an SD of 0.016%. Statistical significance for pair-wise comparisons is shown with ** *p* < 0.01.

In a more detailed analysis, we looked at the role of the different RNA secondary structure domains of the HCV 5′UTR (Figure 4). Here, either the HCV 5′UTR stem-loops I and II including the sequence in between were present in the SLI-II construct (compare Figure 1B), or SLI-III, or the complete 5′UTR with the core protein. The transfected RNAs of all constructs were degraded equally well over time after transfection (Figure 4A). Minus strands again could be detected well with both the SLI-III and the 5′UTR replicons at 48 and 72 hpt (Figure 4B). Thereby, the 5′UTR replicon performed slightly better than the SLI-III construct (significant at 72 hpt). The SLI-II construct performed much worse; however, in the magnification (Figure 4C) we see at 72 hpt that the SLI-II construct actually shows residual replication (about 2% of the WT), which is significantly different from the polymerase-deficient 5′UTR construct. Nevertheless, the SLI-II construct replicates far worse than the SLI-III and 5′UTR constructs (Figure 4B). This indicates that the stem-loop regions I and II of the HCV 5′UTR allow only a very basic replication with extremely low efficiency, and the regions of the SLs III and IV contain important determinants required for replication.

### 2.4. Mutations That Affect Binding of eIF3 and the 40S Subunit to the HCV 5′UTR Impair Replication

According to the results described above, the replicon constructs containing SLI-III or the complete 5′UTR in Gene Cluster I allowed efficient minus-strand production and thereby supported replication, in contrast to the SLI-II construct that shows extremely inefficient replication. Thus, the large stem-loop “domain” SLIII contains determinants that strongly stimulate minus-strand synthesis. In the large SLIII domain, the small sub-domain stem-loops IIIa and IIIb contribute to the binding of eIF3, and the deletion of stem-loop IIIb abolishes the binding of eIF3 without affecting the binding of the 40S subunit [57]. The small stem-loops IIIc, IIId and IIIe are required for the binding of the 40S subunit, and the mutation of either SLIIId or SLIIIe abolishes the binding of the 40S subunit without affecting the eIF3 binding [57].

**Figure 5:**
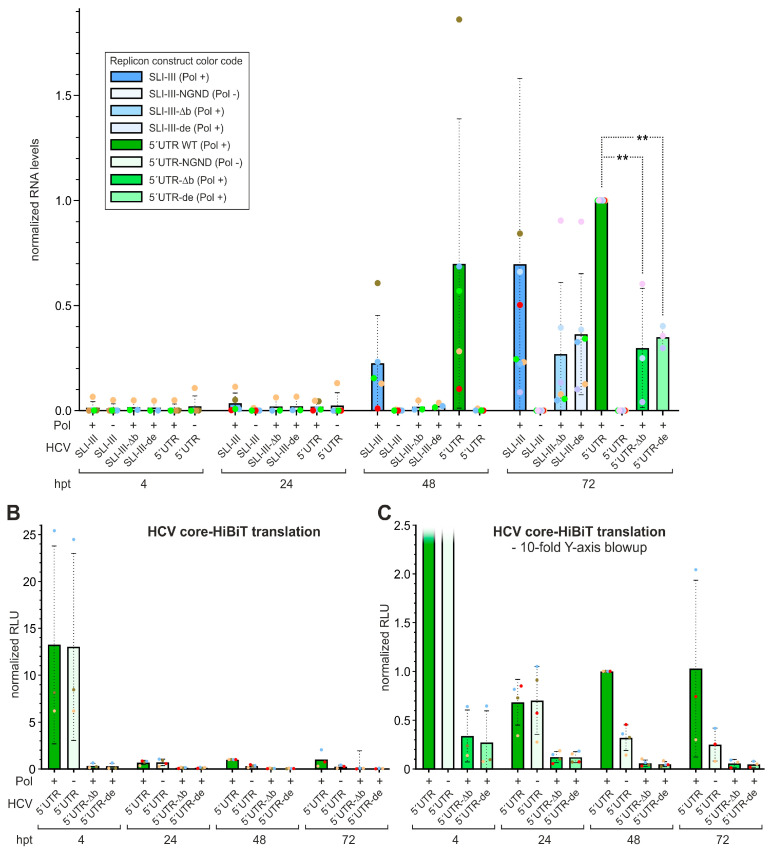
Effect of mutations affecting binding of eIF3 and the ribosomal 40S subunit on minus-strand synthesis. (**A**) Strand-specific RT-qPCR detection of 5EU labeled minus strands. In constructs “Δb” the SLIIIb was deleted (compare Figure 1B, lower panel), which severely affects eIF3 binding. In mutant constructs “de” the SLIIId and SLIIIe were mutated, which severely affects 40S subunit binding. (**B**) Expression of HCV core protein with C-terminal triple HiBiT tags from of the above constructs. This expression is a direct measure for the ability of the respective HCV IRES in Gene Cluster I to mediate IRES-dependent translation. (**C**) 10-fold magnification of Y-axis from (**B**) to show low-value details. Statistical significance for pair-wise comparisons is shown with ** *p* < 0.01.

Therefore, in the SLI-III as well as in the 5′UTR constructs, we applied mutations affecting the eIF3 or 40S binding. We deleted the small sub-domain SLIIIb (construct name appendix “-Δb”) to abolish the eIF3 binding without affecting the 40S subunit binding. Likewise, we mutated both small sub-domains SLIIId and SLIIIe (name appendix “-de”) to abolish the 40S subunit binding without affecting the eIF3 binding (compare Figure 1B, right part of lower panel). Our results in Figure 5A show that deletion of SLIIIb (SLI-III-Δb) reduces minus-strand synthesis to a similar level as the SLI-III-de mutation in both constructs, i.e., to about 50% of WT in the SLI-III context and to about 30% in the 5′UTR context.

As a control, we measured Core-HiBiT expression in the 5′UTR context. As expected, both mutations disable translation directed by the mutated HCV IRES in Gene Cluster I (Figure 5B,C). The magnification of the Core-HiBiT data (Figure 5C) shows that up to 24 hpt the Core-HiBiT expression from Gene Cluster I remains the same when polymerase-proficient (Pol +) and polymerase-deficient (Pol −) 5′UTR constructs are compared. This indicates that up to 24 h after transfection, HCV IRES-directed translation in Gene Cluster I largely reflects the translation of the transfected replicon RNAs and remains largely unaffected by a NS5B polymerase defect. In contrast, after 24 hpt (i.e., at 48 and 72 hpt), the (Pol −) construct’s HiBiT expression strongly declines in comparison to the (Pol +) construct. This indicates that from this time, mainly replicon RNA genomes are detected that are protected in HCV replication complexes and/or produced by “roundabout” amplification.

## 3. Discussion

In this study, we investigated the role of sequences in the HCV 5′UTR in the regulation of minus-strand synthesis that starts at the 3′end of the plus-strand. We found that the complete absence of HCV sequences at the genome 5′end abolishes RNA replication. The mere presence of SLI and SLII (including the two miR-122 binding sites in between) allows only very low-level minus-strand synthesis (about 2% of WT 5′UTR), whereas the constructs SLI-III and the complete 5′UTR allow efficient minus-strand synthesis.

The replicon construct with only an artificial hairpin but no HCV sequences at the 5′end of the plus strand could in principle exhibit minus-strand synthesis completely uncoupled from other processes. This construct shows no replication, and by that it demonstrates that HCV sequences at the plus-strand 5′end or at the minus-strand 3′end are absolutely required for minus-strand synthesis or replication, respectively. However, a limitation of our study (and others) is that whenever we add any genuine HCV sequences at the 5′end of the replicon, “roundabout” amplification is—strictly speaking—no longer excluded. While our replicon system does uncouple minus-strand synthesis from translation, assembly and packaging, it cannot specifically rule out possible effects on positive-strand RNA synthesis. Thus, we face the dilemma that mutations in the annotated plus-strand sequence may either affect the function of the physical plus strand, or they may affect the function of the opposite minus strand (or both). In the above-mentioned case of the SLI-II construct, comparison of RNA synthesis efficiencies from different studies may help; however, we must face the objection that the use of different experimental systems may also contribute to such differences.

Importantly, our mutations in the SLIII domain of the plus strand come with some features that make it rather unlikely that only the function of the opposing minus strand is affected. Those sequences or RNA secondary structure elements in the SLIII domain that bind the translation initiation factor eIF3 (namely, the subdomain SLIIIa and, more important, SLIIIb) are important in this context. The deletion of SLIIIb solely knocks out the binding of eIF3, without affecting the binding of the small ribosomal 40S subunit [57]. Hereby, our precise deletion of the plus strand SLIIIb differs from mutations placed by other groups in the opposing minus-strand 3′region. Mutations affecting the stem base of minus-strand SL-E1 (the approximate mirror of the plus strand SLIIIb) [24] precisely affect or compensate secondary structure features that are unique to the minus strand SL-E1 but not to the plus strand SLIIIb and this proves the presence and importance of these secondary structures in the minus strand. Though approximately a mirror image, the minus-strand SL-E1 still does not represent the entire SLIIIb of the plus strand. The apical loop in the minus-strand SL-E1 is larger, and the upstream sequence of the stem is moved in comparison to its plus-strand SLIIIb counterpart [24], resulting in a different base pairing in the stem. In contrast, our precise deletion of the SLIIIb accurately deletes only the plus-strand SLIIIb (please compare [57]) and leaves the remainder of the SLIII’s apical four-way junction intact; this is the mutation that precisely knocks out the eIF3 binding [57].

Similarly, the mutation of sub-domain stem-loops SLIIId and SLIIIe solely impairs the binding of the 40S subunit, without affecting the eIF3 binding [57]. Here, we have precisely mutated each of the three nucleotides in the apical loops of the stable small stem-loops SLIIId and SLIIIe that are embedded in a well documented overall RNA secondary and tertiary structure setting of the plus-strand SLIII domain and were shown in many studies to precisely knock out the 40S subunit binding and other functions that depend on precise RNA structure [30,31,57,58,59,60,61].

Importantly, the functional effect of these mutations on minus-strand synthesis is the same as that of the SLIIIb deletion in both the SLI-III and the 5′UTR construct, although the structural features of these two types of mutations as well as their effects on the binding of eIF3 or the 40S subunit, respectively, are completely different. This strongly argues for their function in the plus strand but not in the minus strand. Moreover, the opposing minus-strand sequences (i.e., upstream of the minus-strand SL-E1) appear rather volatile in their predicted structures [2,19,20,21,22,23,24]. Therefore, we conclude that the sequences in the plus-strand SLIII domain that bind eIF3 and the 40S subunit are essential for efficient minus-strand synthesis.

In other studies using replicon systems, the SLI-II region and the SLIII region were shown to be important for replication [12,14,16], with a tendency for a greater importance of the SLI-II region, and some differences between the studies that likely depend on replicon design as well as methods and times of output measurements. Thus, we are in agreement with the above studies that SLIII region sequences are also very important for replication, while our study places greater emphasis on the importance of the SLIII sequences. Importantly, going beyond the above studies, we show here that two different types of small mutations that each specifically recruit one particular translation machinery component (either eIF3 or the 40S subunit) reduce replication efficiency to about 40–50%. Thus, it appears as if we have hit—with both different types of mutations—a kind of “master regulator” function in the plus-strand’s SLIII domain that is involved in HCV RNA 5′-3′end communication via eIF3 and the 40S subunit.

Our results are consistent with the findings from the laboratory of Jennifer Doudna that the 40S subunit and eIF3 each bind with quite similar affinities to the HCV IRES or the 3′UTR. The isolated 40S subunit binds to the 3′UTR [62] with a K_D_ of about 3.5 to 6.7 nM [63], which is only slightly higher than the K_D_ of 2.0 nM for the binding to the IRES [57]. The K_D_ of the binding of eIF3 to the HCV 3′UTR was found to be about 8.9 nM [63], which is lower than the K_D_ of 35 nM for the binding of eIF3 to the IRES [57]. Accordingly, the combined 40S-eIF3 complex has a K_D_ of 1 nM for its binding to the 3′UTR [63], which is the same as for its binding to the IRES [57]. Also, for other plus-strand RNA viruses, the interaction of eIF3 and the 40S subunits with the 3′- and 5′-UTR was shown. In Barley Yellow Dwarf Virus (BYDV) RNA, both the 5′UTR and a cap-independent translation enhancer element (CITE) in the 3′UTR bind eIF3 and the 40S subunit with similar affinities [64]. The 3′UTR of West Nile Virus (WNV) directly recruits ribosomal 40S subunits to stimulate translation [65]. However, these above studies focused on the regulation of translation, not replication. Nevertheless, it is tempting to speculate if the binding of translation initiation components to the 3′UTR may not only regulate translation but also the replication of these viruses and perhaps also of other plus-strand RNA viruses.

Some hypotheses about the events taking place at the ends of the HCV genome as well as their implications for the HCV life cycle have been proposed, but the situation still appears to not be completely understood. Here, we suggest a hypothesis that combines as many of the facts as possible. As detailed above, both the 40S subunit and eIF3 bind both the IRES and the 3′UTR with the same high affinity [57,63], making 40S and eIF3 the most prominent candidates for triggering a 5′-3′-end interaction. Partial hybridization of the genome ends may support this circularization [2].

In vitro assays for the binding of miR-122 to both target sites in the 5′UTR in the absence of Ago proteins revealed a K_D_s between 11 and 900 nM for both sites, or even much higher for site 2, depending on the flanking sequences and the conditions in the study [66,67]. In cells, the binding of both miR-122/Ago2 complexes occurs cooperatively [68,69], and Ago2 protein (initially described as “eIF2C” from ribosomal salt wash) can routinely interact with ribosomes [70]. The 3′UTR also contains a conserved miR-122 binding site [2]. The binding of miR-122 to the 5′UTR shapes the IRES towards the formation of SLII and thus to the translation-active conformation [71,72], thereby supporting the 40S binding. The NS5B polymerase likely acts as a dimer or oligomer, and NS5B appears to make multi-point interactions with the CRE, the polyU, its actual start site at the 3′end [29,34,43,44,45], and NS5B can also bind ribosomes [73]. Moreover, the binding of NS5B to the HCV 5′end is stimulated by miR-122 [67], and the K_D_ of the NS5B-5′UTR interaction is by far lower than the K_D_ of the interaction of Poly(rC) Binding Protein 2 (PCBP2) with the 5′UTR [67].

PCBP2 has three RNA-binding domains and multimerizes [74,75]; it can bind to the SLI of the 5′UTR [76,77], to the C-rich seed sequence of the second miR-122 binding site upstream of SLII [78], and more weakly to the 3′UTR [76,77,79], and it can induce HCV RNA genome circularization in vitro [67,77]. The binding of miR-122 and PCBP2 at the 5′end appears mutually exclusive [67,78], but nevertheless miR-122 stimulation of viral RNA synthesis was reported to be PCBP2 dependent [78]. In in vitro assays, PCBP2 competes with the NS5B binding to the HCV 5′UTR 5′-terminus [67], and PCBP2 limits HCV genome packaging [80].

**Figure 6:**
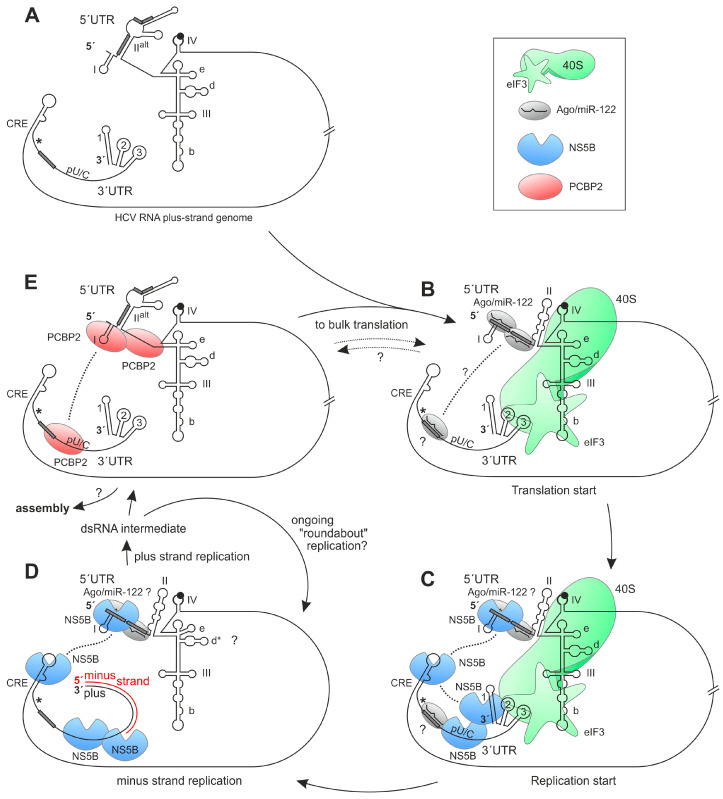
Model of the possible interactions of 40S ribosomes, eIF3, miR-122/Ago complexes, NS5B and PCBP2 with the HCV 5′- and 3′-UTRs. (**A**) The HCV plus-strand RNA with the 5′- and 3′UTRs. In the 5′UTR, the SLII and the region between SLI and SLII are refolded to an alternative version of SLII, SLII^alt^, in which both miR-122 binding sites are covered. The black dot indicates the polyprotein start codon. (**B**) Binding of two miR-122/Ago complexes to the 5′UTR, refolding of SLII, and binding of 40S subunit and eIF3 to both UTRs starts the pilot round of translation. (**C**) After the first ribosomes have translated the polyprotein, NS5B dimers or oligomers (in replication complexes with other replication proteins, including the protease/helicase NS3, not shown here) bind in multi-point interactions to the CRE, polyU, 3′end and the 5′end with miR-122/Ago complexes, and NS5B starts minus-strand synthesis at the 3′end of the plus strand. (**D**) NS5B synthesizes the minus strand on the plus-strand template; other factors are likely displaced from the RNA. The small stem-loop d* may form as an alternative secondary structure in the large stem-loop domain III upon ribosome release. (**E**) PCBP2 binds to the 5′UTR and 3′UTR and maintains genome circularization. For details, please see main text and also compare Figure 1A.

Thus, there appear to exist at least four different states of the HCV RNA in complex with factors (Figure 6). In state 1 (Figure 6B), miR-122/Ago complexes bind to the 5′UTR (without PCBP2) and perhaps to the 3′UTR, and the IRES folds into the translation-competent state with SLII and binds the ribosomal 40S subunit and eIF3. In this state, the pilot rounds of polyprotein translation are started. In state 2 (Figure 6C)—after the first ribosomes have completed polyprotein translation—NS5B binds in a multi-point interaction with the CRE, the polyU, the 3′end and the 5′UTR, with the latter being stimulated by miR-122.

It is not clear if the binding of Ago2/miR-122 complexes to the 3′UTR still occurs simultaneously or if they are released upon the NS5B binding in this state, but a transient interaction of miR-122/Ago complexes, NS5B, eIF3 and the 40S subunit on the RNA might explain how miR-122 actually stimulates HCV RNA replication [81].

This multi-point interaction appears to be required to support the initial start of minus-strand synthesis, since the 3′end of the plus strand is a much worse template for the NS5B polymerase than the 3′end of the minus strand [41]. This, in turn, may contribute to the selectivity of NS5B for its genuine template (as outlined above).

In state 3 (Figure 6D), NS5B uses the plus strand as a template to synthesize the minus strand, and ribosomes, eIF3, miR-122/Ago complexes and other factors are displaced from the RNA. From this state, the minus strand is used as a template for the synthesis of progeny plus strands, resulting in the double-strand (ds) RNA intermediate. It is likely that the genome then undergoes repeated rounds of “roundabout” amplification. From that, progeny plus strands can be used for the assembly of new virus particles. Alternatively, the progeny plus strands can be used for the bulk translation of viral proteins (Figure 6B) to yield sufficient structural proteins for assembly. State 4 in between (Figure 6E) has a dimer of PCBP2 bound to the SLI in the 5′UTR, connecting it with the 3′UTR and circularizing the genome. The functional logic of states 1, 2 and 3 appears clear, but what is the function of state 4? Does the binding of PCBP2 just serve to limit the exit of genomes towards the assembly [80] before enough structural proteins have accumulated, and is there a competition between the binding of PCBP2 (Figure 6E) and the binding of miR-122/Ago and translation components (Figure 6B) and thus between the fate of the RNA genome to undergo either further bulk translation or assembly?

The above scenario could also be compatible with a contribution of NS5B to the selection of the HCV RNA genome for encapsidation (packaging) into assembling virus particles. Until now, not a single “encapsidation signal” was identified; however, the specificity of HCV RNA genome encapsidation may be accomplished by a combination of determinants or events, respectively. In comparison, in Hepatitis B Virus (HBV), a single clearly defined RNA signal is necessary and sufficient for specific RNA pregenome encapsidation [82], and it interacts specifically with one molecule of the viral polymerase [83]. In contrast, in Human Immunodeficiency Virus (HIV), encapsidation of the viral RNA genome is accomplished by its interaction with the more abundant gag capsid protein, but it is likely that a kinetic component is involved in rendering packaging selectivity [84]. In contrast, in the HCV genome, several *cis*-acting signals were described to contribute to encapsidation, like the 3′UTR [85] and some small stems with G-rich loops that are scattered over the RNA genome and interact with the core protein [86]. Perhaps both specific RNA *cis*-determinants and spatial and kinetic aspects act together. The HCV RNA plus strand is specifically recognized by the above-described multi-point interaction of NS5B with the CRE, polyU and 3′X. During replication, the progeny plus strands may slide like they are on a railway formed of NS5A and NS5B in order to reach the core proteins in the protected environment of the replication organelles [87], where finally the small stems with G-rich loops scattered over the RNA genome tether the RNA to the core proteins inside the growing particle. In total, this perhaps provides enough specificity for a selective incorporation of HCV RNA genomes into the assembling virus particles, without a further need for “the one” single, required and sufficient RNA–protein recognition event as occurs in HBV.

## 4. Materials and Methods

**Replicon plasmids:** The so-called 4th generation replicon RNA sequence contains three functionally independent Gene Clusters (Figure 1B). Gene Cluster I contains the HCV 5′UTR sequences which are under investigation for their possible function in the stimulation of minus-strand RNA synthesis. Alternatively, only a hairpin is used as the negative control. Gene Cluster I is described with its variations in detail below. Gene Cluster II contains an independent protein expression cassette with the *Firefly* luciferase (Fluc) gene [88] under translational control of the Poliovirus IRES (GenBank: V01149.1, position 117–742). Gene Cluster III contains two principal regions: (i) the IRES of Encephalomyocarditis virus, EMCV (GenBank: NC_001479.1, pos. 263–830) fused by the sequence ACCATG to the downstream HCV NS3 to the NS5B replication protein coding sequence (GeneBank: AB047639, pos. 3431–9442), and (ii) the HCV 3′UTR, which serves as the start point for minus-strand RNA synthesis (pos. 9443–9678) [89].

Downstream of this replicon RNA sequence follows a Hepatitis Delta Virus (HDV) genomic ribozyme sequence (GeneBank: M21012.1, pos. 686–772) that serves to generate an authentic 3′end of the HCV 3′UTR. The HDV ribozyme is followed by a phage T7 terminator sequence (GeneBank NC_001604.1, pos. 24164–24210) to avoid run-through transcripts from plasmid templates that might have escaped HDV ribozyme cleavage or linearization at the downstream EcoRI cleavage site. In selected constructs, a wild-type (WT) construct with functional NS5B RNA-dependent RNA polymerase (RdRP or “replicase”) and a polymerase-defective variant were each made. The defective construct had not only the GDD motif in the active center mutated to GND [90] (with D318 mutated to N) but also the upstream D220 mutated to N [90]; these substitutions inhibit polymerase function by preventing the essential binding of Mg^2+^ ions. This type of polymerase-defective construct was named “NGND” or “Pol −” for short.

Gene Cluster I comes in different variations (see Figure 1B, lower panel). Replicon RNA in vitro transcription is started from a T7 promoter upstream of Gene Cluster I. In this T7 promoter sequence (TAATACGACTCACTATAG), the last G is the first transcribed nucleotide. In Gene Cluster I, the basic negative control “hp” construct contains a 5′-terminal “hairpin” stem-loop structure with a stem of 19 uninterrupted base pairs (bp) (74% GC content; including the first transcribed G) and a tetraloop for providing stability against exonucleases. This hairpin is followed by a 220 nucleotide (nt) spacer. In all constructs containing HCV 5′UTR sequences, the last G of the promoter sequence replaces the first authentic HCV nucleotide (A → G) of the Jc1 chimeric isolate [89] for higher transcription efficiency; in natural HCV isolates this first nucleotide can be G > A > U [2]. Moreover, the spacer was reduced to about 160 nts. In the construct SLI-II, HCV sequences from pos. 2 to 117 replace the hairpin of the basic “hp” construct. Together with the A → G exchange of the promoter sequence, these nucleotides represent pos. 1 to 117 of the HCV 5′UTR, ending at the last nucleotide of stem-loop II [10]. In the SLI-III construct, the HCV sequences extend to pos. 329; thus, this construct contains all SL I, II and III sequences, ending just upstream of the SLIV. In the HCV 5′UTR construct, the entire HCV 5′UTR is present along with the sequence coding for amino acids (AA) 1 to 165 of the HCV core protein. This avoids the C-terminal core protein cleavage sites and the hydrophobic membrane anchor [91,92]. Three copies of the HiBiT tag [93], each preceded by 10 AA Gly/Ser-linkers, are fused to the core C-terminus.

In both the SLI-III and the 5′UTR constructs, each two types of mutations were applied that impair the binding of eIF3 or the small ribosomal 40S subunit to the HCV IRES [57]. In the SLI-III ΔIIIb construct (in short “Δb”) the apical stem-loop IIIb of the SLIII domain which binds eIF3 [57,94] is replaced by a small unrelated stem-loop sequence (AATTGCCATT), while the mutIIId/e construct (in short “de”) contains two mutations both in stem-loop regions IIId (GGG > CCC) and IIIe (GAUA > GAAA) which impair the 40S subunit binding [57].

**Reagents:** Enzymes: T7 RNA polymerase (New England Biolabs (NEB), Frankfurt am Main, Germany; M0251L); DNase I (RNase free) (NEB M0303S); TURBO DNase (Thermo Fisher Scientific, Waltham, MA, USA; AM2238); Maxima Reverse Transcriptase (Thermo Fisher EP0743). Kits: Click iT Nascent RNA Capture Kit (Invitrogen, Life Technologies GmbH, Darmstadt, Germany; C10365); Monarch RNA isolation kit (NEB T2040L); LunaScript RT Master Mix Kit (Primer-free) (NEB E3025L); Luna Universal qPCR Master Mix (NEB M3003); Steady-Glo Luciferase Assay System (Promega, Walldorf, Germany; E2510); Nano-Glo HiBiT Lytic Detection System (Promega; N3030).

**Non-standard chemicals:** Lipofectamine MessengerMax Transfection reagent (Invitrogen; LMRNA008); Acidic phenol/chloroform/isoamylalcohol (PCI) (125/24/1, pH 4.5, Thermo Fisher; AM9722); Biotin-X-azide (carboxamide-6-azidohexanyl biotin) (Lumiprobe Life science solutions, Hannover, Germany; 2730-100 mg).

**Biological Resources:** Huh-7.5 cells [95]; Hepatitis C Virus clone Jc1 [89]; Poliovirus IRES [96]; Firefly luciferase [88]; EMCV IRES [97].

**In vitro transcription and RNA purification:** The 11955 nt RNA corresponding to the 4th generation replicon system was always freshly synthesized before transfection into Huh-7.5 cells. The plasmid template DNA was linearized prior to in vitro transcription with EcoRI. The in vitro transcription reactions included the buffer provided by the supplier, plus 5 mM of additional MgCl_2_, 4 mM rNTPs, 10 mM DTT, 0.5 µL Murine RNase Inhibitor, 2 µL of T7 polymerase and 1 µg of template DNA in a total volume of 50 µL and were incubated at 37 °C for 4 hrs. After in vitro transcription, the template DNA was digested by adding 2 µL of TURBO DNase I (NEB) and incubating for 30 min at 37 °C. The RNA was purified using a Monarch column (NEB) and eluted in water. TURBO-DNase digest and column purification were then repeated.

**Transfection of RNA by Lipofection:** RNA was transfected into Huh-7.5 cells at ~90% confluency in 6-well or 12-well plates. The lipofection reagent, Lipofectamine MessengerMax (ThermoFisher, LMRNA008), was used at a 3:1 ratio (3 µL for 1 µg of RNA). RNA and Lipofectamine were first mixed separately with Opti-MEM and then combined to make one transfection solution. To this end, for each well, 3.5 µg RNA (6-well) or 1.9 µg (12-well) was thoroughly mixed with 100 µL of Opti-MEM by gentle vortexing. In another vial, the appropriate amount of Lipofectamine was mixed with 100 µL of Opti-MEM for each well and mixed by vortexing. Both solutions were combined after 5 min of incubation at room temperature and incubated for a further 15 min. Meanwhile, the Huh-7.5 cells were washed with 1× PBS, and 800 µL Opti-MEM (6-well) or 300 µL (12-well) was gently added to the cells. At this point, 0.1 mM of 5-Ethynyl Uridine (5EU) was pre-mixed with the Opti-MEM if a nascent RNA capture assay was planned. The transfection solution was added dropwise to the cells, and the plates were gently rocked back and forth to ensure complete mixing of all reagents. The cells were incubated at 37 °C. The medium was replaced by complete medium after 4 h.

**Total RNA Extraction using TRIzol:** Total RNA was extracted from the Huh-7.5 cells at certain time points (4, 24, 48 and 72 hpt). After the medium was removed from the cells, 1 mL of TRIzol was added directly to the cells and incubated for 5 min at room temperature. The lysed cells were mixed thoroughly in TRIzol by pipetting up and down several times and transferred to 1.5 mL sterile tubes. A total of 200 µL chloroform was added to the lysate and mixed properly by inverting the tubes 20 times. The solution was incubated for a further 3 min at room temperature and then centrifuged at 14,000 rpm for 15 min at 4 °C. The upper aqueous phase contained RNA as well as traces of residual genomic DNA. Approximately 400–500 µL of the upper phase was transferred to new tubes, carefully avoiding the interphase.

Extending the standard RNA purification procedure using TRIzol, again 200 µL chloroform was added and mixed thoroughly by inverting the tubes 20 times to remove residual traces of phenol. The solution was centrifuged at 14,000 rpm for 5 min at 4 °C and the upper aqueous phase was transferred to new tubes. An equal volume of ice-cold isopropanol was added along with 1.5 µL of GlycoBlue (15 mg/mL), mixed well and incubated at −20 °C overnight. RNA was pelleted by centrifugation at 14,000 rpm for 30 min at 4 °C. The pellet was washed twice with 70% ethanol, air-dried for 10 min, and gently dissolved in 30 µL of deionized water.

At this stage, commonly used standard RNA purification procedures end. However, such RNA preparations still contain traces of genomic DNA and also residual transfected plasmid DNA as contamination. Therefore, 2 µL DNase I (NEB) was added to the RNA along with 10× DNase I buffer, 0.5 µL murine RNase Inhibitor and water up to 100 µL. Reactions were incubated at 37 °C for 30 min. The mixture was then diluted to 200 µL, and RNA was extracted using acidic phenol (Phenol/Chloroform/Isoamyl alcohol; 125:24:1; pH ~ 4.5; ThermoFisher). Separation of DNA from RNA is then based on their differential solubility in the aqueous and phenol phases at specific pH levels [98]. Both DNA and RNA are soluble in the aqueous phase when the pH is around 6 to 7. At lower pH, phosphate backbones are more protonated, and DNA can partition into the more hydrophobic phenol phase. In contrast, RNA remains largely in the aqueous phase due to the additional hydrophilic 2′-hydroxyl group. This separation works best with high (5/1) phenol/chloroform ratios, where the presence of residual water in the phenol phase [99], in combination with the low concentration of hydrophobic chloroform, provides a largely hydrophobic but still slightly hydrophilic environment for the DNA. The RNA was then collected from the upper aqueous phase by standard ethanol precipitation and dissolved in water.

To ensure complete elimination of contaminating DNA, the DNase treatment was then repeated, this time using 2 µL of TURBO DNase along with the appropriate buffer and 0.5 µL of murine RNase Inhibitor in a final volume of 50 µL. Finally, the RNA was additionally purified using silica-based Monarch kit (NEB) columns according to the manufacturer’s instructions. The purified RNA (virtually free of contaminating DNA) was eluted in 20 µL of water. The amount of RNA was determined using a Qubit reader, and integrity was checked by agarose gel electrophoresis.

**Reverse transcription and quantitative PCR reactions:** RT-qPCR was applied for the strand-specific quantification of the RNA synthesized by the HCV replicon system inside the Huh-7.5 cells. All primers for plus- and minus-strand detection target different parts of the EMCV IRES region which is artificial for eukaryotic cells and thus unique for the replicon system. For HCV plus-strand RNA as well as GAPDH mRNA detection, 1 µg of RNA in 7 µL water was heated to 70 °C for 5 min. Meanwhile, 10 µM RT primer “D_TAG_RT_plus_1” (gcaggagctaagcgctggTcaggagctaagAAGACCCCTAGGAATGCTC-GTCAAGAAGACAGGGCC) was also preheated at 70 °C. After 5 min, 1 µL of primer was added to the RNA, keeping reactions constantly at 70 °C on a heating incubator. The temperature was then decreased to 65 °C without removing the tubes from the incubator. During the temperature drop, 2 µL of LunaScript RT Master Mix Kit (Primer-free) (which includes LunaScript Reverse Transcriptase with reduced RNase H activity) was added to the tubes to reach a total volume of 10 µL and mixed by pipetting up and down. The RT reaction for plus-strand detection was then performed at 65 °C for 20 min, followed by inactivation of the reverse transcriptase at 95 °C for 1 min. RT for GAPDH was performed at 55 °C for 20 min, followed by RT inactivation.

For the plus-strand qPCR reaction, 2 µL of 1:10 or 1:100 cDNA dilution was mixed with 10 µL of Luna Universal qPCR Master Mix (which includes Hot Start Taq DNA Polymerase), 0.1 µM forward primer “Amp_1_plus_F” (CGAAGCCGCTTGGAATAAGGCC-GGTGTG) and 0.2 µM reverse primer “D_TAG “ (gcaggagctaagcgctggTcaggagctaag) in a final reaction volume of 20 µL; alternatively, all volumes were scaled down to reach a final reaction volume of 10 µL. The reaction mix was transferred to 96-well plates which were sealed immediately to prevent any contamination. The qPCR program for plus-strand detection was as follows: initial denaturation at 94 °C for 60 s; then 40 cycles of denaturation at 94 °C for 30 s and annealing/elongation at 62 °C for 30 s, followed by a final melting curve analysis at 85 °C. For GAPDH detection, primer “GAPDH 2_rev” (ACCACCCTGTTGCTGTAGCCAA) was used as RT primer and qPCR reverse primer, “GAPDH 2_for” (GTCTCCTCTGACTTCAACAGCG) as qPCR forward primer; the qPCR parameters were the same as before except that annealing was performed at 55 °C. RT-qPCR for minus-strand detection was performed as described in the following nascent RNA capture assay.

**Nascent RNA Capture Assay:** The nascent RNA capture assay allows the screening of newly synthesized RNA from the whole content of the total RNA present inside the Huh-7.5 cells. In conventional transfection and RNA extraction protocols, the RNA extracted from the cells contains both the newly synthesized minus-strand RNA produced after transfection of the in vitro transcribed HCV replicon RNAs, as well as all pre-existing plus strands and other RNA (including residual undegraded template DNA) that were transfected to the cells. However, some of the transfected HCV plus-strand RNA itself remains functional inside the cells even after 120 hpt and increases the RT-qPCR signals drastically, even in the negative controls with replication-deficient constructs. A large part of these false positive signals may be due to partially degraded RNA oligonucleotides that act as unwanted primers in the RT reaction.

To overcome this problem, the nascent RNA is labeled using modified uridine molecules (5-Ethynyl-Uridine, 5EU) which are added to the cell culture medium only at the time of transfection. In this way, only RNA newly synthesized after transfection is labeled with 5EU, while the bulk of pre-existing RNA in the cell remains unlabeled, as well as the transfected RNA and all DNA in the cell. Total RNA is extracted from the cells by TRIzol extraction and further purification. In the total RNA preparation, an azide-modified biotin molecule is then covalently bound to the incorporated 5EU through a copper-catalyzed click reaction. The biotin-labeled RNA can then be specifically captured using streptavidin magnetic beads, extensively washed, and immediately used for strand-specific detection via RT-qPCR, while all non-5EU-labeled RNAs and DNA are efficiently removed during the washing steps. All necessary reagents for the assay were provided in the “Click iT Nascent RNA Capture Kit”.

**Nascent RNA labeling with 5EU:** 0.1 mM of 5EU was added to the Opti-MEM at the time of lipofection. After 4 h, the Opti-MEM was replaced with complete medium supplemented with 0.2 mM 5EU. The same amount of 5EU was additionally supplemented every 24 h.

**EU-Total RNA extraction:** After the Total RNA Extraction using TRIzol (see above) at specific time points (4, 24, 48 and 72 hpt) and all further DNase digestion and purification steps, the total EU-RNA was eluted from the Monarch columns in 20 µL of water. The concentration was determined using a Qubit Fluorometer, and the integrity was checked by gel electrophoresis. RNA was stored at −20 °C until further use.

**Biotinylation:** Purified total RNA including the 5EU-labeled RNA (8–10 µg) was mixed with 0.5 mM Biotin-X-azide, 2 mM CuSO_4_ and 25 µL Click-iT EU buffer (Component B) to prepare a final 50 µL reaction cocktail. To this cocktail, 1.25 µL of Click-iT reaction buffer additive 1 (Component E) was added and mixed immediately by pipetting up and down. This marked the initiating point of the click reaction between 5EU and biotin-azide. After 3 min, 1.5 µL Click-iT reaction buffer additive 2 (Component F) was added, which caused the solution to turn dark brown. The click reaction was incubated at room temperature with agitation at 300 rpm for 30 min.

The procedure was followed by extraction. To the click reaction, 700 µL chilled ethanol (100%) and 75 µL of 5 M ammonium acetate were added to precipitate all the RNA (including the biotinylated EU-RNA) at −40 °C overnight. The RNA was pelleted by centrifugation at 14,000 rpm for 20 min at 4 °C. The supernatant was removed, and the pellet was washed twice with 70% ethanol. The RNA was air-dried for 10 min and finally dissolved in 20 µL of deionized water. The concentration was determined by Qubit Fluorometer. Biotinylation did not significantly affect total RNA yield; approximately 90% of the starting material was recovered. The RNA was stored at −20 °C.

**Purification of 5EU-labeled RNA with Streptavidin Magnetic Beads:** Before binding, 48 µL aliquots of streptavidin magnetic beads per vial were immobilized on a magnetic rack, the supernatant was removed, and the beads were resuspended in 480 µL of Component J. The beads were immobilized again on the magnetic rack for 1 min, and the supernatant was discarded. The washing step was repeated another two times. Finally, the beads were resuspended in 48 µL of Component J. The RNA binding reaction was prepared by adding 31 µL of Click-iT RNA Binding Buffer (Component G) to 1 µg of the RNA preparation including the biotinylated EU-RNA, along with 0.5 µL of murine RNase inhibitor (NEB), and deionized water added to a final volume of 62 µL. The reactions were heated to 70 °C for 5 min. Next, each 12 µL of the streptavidin magnetic beads, previously washed and resuspended in Component J, were immediately added to each RNA binding reaction, mixed thoroughly by pipetting up and down, and the tubes returned to room temperature. To facilitate the binding of biotinylated EU-RNA to the beads, the reaction tubes were agitated at 300 rpm at room temperature for 30 min.

The streptavidin magnetic beads with the bound 5EU-labeled RNA were then washed ten times to remove contaminating non-biotinylated RNA. The beads were immobilized on a magnetic rack for 1 min, the supernatant was discarded, and the beads were thoroughly resuspended in 120 µL of Click-iT Reaction Wash Buffer 1 (Component I). The beads were immobilized again for 1 min, and the supernatant was removed and replaced by fresh 120 µL of Component I. The wash step was repeated for a total of 5 times using Component I. The washing procedure was then repeated an additional five times using 120 µL of Click-iT Reaction Wash Buffer 2 (Component J). Finally, the beads were resuspended in 12 µL of Component J and immediately used for the reverse transcription reaction.

**Reverse Transcription and qPCR reactions for minus-strand detection:** 12 µL of RNA bound streptavidin beads, resuspended in sterile Click-iT reaction Wash Buffer 2 (Component J), were diluted to a final volume of 15 µL in 0.2 mL DNase-/RNase-free PCR tubes and heated up to 70 °C for 5 min. Meanwhile, the RT primer “D_TAG_RT_minus_2” (gcaggagctaagcgctggTcaggagctaagGTAGCGACCCTTTGCAGGCAGCGGAACC) was also preheated at 70 °C. After 5 min, 1 µL (10 µM) of the preheated primer was added to the bead suspension while maintaining the temperature at 70 °C. Subsequently, the temperature was reduced to 65 °C, without removing the tubes from the incubator. During the temperature drop, 4 µL of LunaScript RT Master Mix (Primer-free) (including LunaScript Reverse Transcriptase) was added to the reaction and mixed by pipetting up and down. The reverse transcription reaction was performed at 65 °C for 30 min with gentle agitation at 300 rpm. Following the reaction, the PCR tubes were transferred to a thermal cycler for enzyme inactivation at 95 °C for 1 min, followed by a hold at 4 °C. The beads were then centrifuged briefly, and 10 µL of the cDNA in the supernatant was carefully transferred to fresh, sterile 0.2 mL tubes for downstream applications.

For the minus-strand qPCR reaction, the cDNA synthesized in the previous step was immediately used by dilution at a ratio of 1:10 with water, and mixed thoroughly with 10 µL of Luna Universal qPCR Master Mix (which includes Hot Start *Taq* Pol), 0.1 µM of forward primer “D_TAG” (gcaggagctaagcgctggTcaggagctaag) and 0.2 µM of reverse primer “Amp_2_minus_R” (ACGTGGCACTGGGGTTGTGCCG) in a final reaction volume of 20 µL. The reaction mix was transferred to 96-well plates which were sealed instantly to prevent contamination. The qPCR program is as follows: initial denaturation at 94 °C for 60 s, then 39 cycles of denaturation at 94 °C for 30 s and annealing/elongation at 62 °C for 30 s, followed by a final melting curve analysis at 85 °C.

**Firefly Luciferase Assay:** The reagent (Steady-Glo Luciferase Assay System) was prepared by adding lysis buffer to the lyophilized luciferin substrate and mixed thoroughly. Protein expression was measured usually at 4, 24, 48 and 72 hpt. The cells in 6-well plates were washed with 1× PBS, and 120 µL *firefly* luciferase assay reagent was added dropwise directly onto the cells. The cells were incubated in the dark at room temperature for 10 min. After that, the cells were detached from the bottom using an appropriate cell scraper, and the whole lysate was transferred to a transparent tube for measurement in a Bethold Lumat LB 9501 luminometer.

**HiBiT Assay:** Expression of viral proteins inside Huh-7.5 cells was measured by bioluminescence using the Nano-Glo HiBiT Lytic Detection System (Promega), which is based on a genetically engineered split Nanoluciferase system [93]. The system requires an 11-amino acid sequence tag (“HiBiT”-tag) fused to the protein of interest. This HiBiT-tag binds with a high affinity to “LgBit”, the large part of Nanoluciferase, constituting a bright luminescent “NanoBit” enzyme. The bioluminescence emitted by the reaction is measured by a luminometer. In some versions of the HCV replicon system used here, a triple HiBiT was fused at the C-terminus of the truncated core protein. The HiBiT lytic buffer was prepared by adding 1:50 volume of HiBiT substrate and 1:100 volume of LgBiT protein at room temperature. The medium was removed, and the cells were washed with 1× PBS. The HiBiT lytic buffer mixture was added dropwise directly onto the cells (120 µL for 6-well and 60 µL for 12-well plates) and incubated in the dark at room temperature for 10 min. The cells were detached from the bottom using a scraper, and the whole lysate was transferred to transparent tubes for bioluminescence measurement.

**Data processing:** The Ct values obtained from RT-qPCR curves (using the Bio-Rad CFX Maestro Software (www.bio-rad.com/de-de/product/cfx-maestro-software-for-cfx-real-time-pcr-instruments; Version 1.0, accessed on 26 February 2018) for Real-Time PCR) were first normalized to the corresponding expression value obtained with GAPDH mRNA (∆Ct = Ct [sample] − Ct [GAPDH]). Then, all these log_2_-scale ∆Ct values were transformed to linear scale 2^−∆Ct^ values. Then, we applied “day-to-day” normalization, i.e., all experimental values of all constructs of one day’s experiment were divided by the value obtained with the WT in that experiment (usually 5′UTR WT at 72 hpt). As a result, all values in a day’s experiment were normalized to its WT, with the WT = 1.0. After this day-to-day normalization, mean and standard deviation (SD) values were calculated for the various constructs from all similar experiments performed on different days. Raw data processing and calculations were usually performed in Microsoft Excel. Statistical significance for pair-wise comparisons was assessed using a Mann–Whitney U-Test in GraphPad Prism software version 8.0.0 for Windows, GraphPad Software, San Diego, CA, USA, www.graphpad.com (* *p* < 0.05; ** *p* < 0.01; *** *p* < 0.001; **** *p* < 0.0001), with *p*-values > 0.05 considered not significant (n.s.). The relative light units (RLUs) obtained from the *firefly* luciferase and HiBiT assay measurements were first corrected by subtraction of the corresponding mock values. Then, RLUs were day-to-day-normalized to the expression level of the 5′UTR at 72 hpt unless stated otherwise, including statistical analyses as described above.

**Novel Programs, Software, Algorithms:** none.

**Web Sites/Data Base Referencing:** Vienna RNAalifold WebServer (http://rna.tbi.univie.ac.at/cgi-bin/RNAWebSuite/RNAalifold.cgi, accessed on 29 March 2026) with the new RNAalifold with RIBOSUM scoring [100]. For visualization of predicted RNA structures, RNA sequences and Vienna dot-bracket outputs from RNAalifold [100] were loaded into VARNA v3-93 (https://varna.lisn.upsaclay.fr/, accessed on 29 March 2026) [101].

## Data Availability

The original contributions presented in this study are included in the article. Further inquiries can be directed to the corresponding author.

## Abbreviations

The following abbreviations are used in this manuscript:

3′UTR	3′Untranslated Region
3′X	highly conserved terminal part of the HCV 3′UTR
5′UTR	5′Untranslated Region
40S	small ribosomal 40S subunit
5BSL3.*	Stem-Loop in the NS5B coding region
Ago	Argonaute protein
CRE	*Cis* Replication Element
eIF3	eukaryotic (Translation) Initiation Factor 3
EMCV	Encephalomyocarditis Virus
Fluc	*Firefly* luciferase
HBV	Hepatitis B Virus
HCV	Hepatitis C Virus
HDV	Hepatitis Delta Virus
HiBiT	An 11 amino acid peptide complementing a defective Nanoluciferase
IRES	Internal Ribosome Entry Site
K_D_	Dissociation constant
NS3	Non-Structural Protein 3
NS5B	Non-Structural Protein 5B
PCBP2	poly(rC) Binding Protein 2
polyU	poly uridine tract
polyA	poly adenylate tract
qPCR	quantitative (Real-Time) Polymerase Chain Reaction
RT	Reverse Transcriptase or Reverse Transcription
SL	stem-loop
